# Fragment-Based Interrogation of the 14–3–3/TAZ Protein–Protein Interaction

**DOI:** 10.1021/acs.biochem.4c00248

**Published:** 2024-08-22

**Authors:** Blaž Andlovic, Dario Valenti, Federica Centorrino, Francesca Picarazzi, Stanimira Hristeva, Malgorzata Hiltmann, Alexander Wolf, François-Xavier Cantrelle, Mattia Mori, Isabelle Landrieu, Laura M. Levy, Bert Klebl, Dimitrios Tzalis, Thorsten Genski, Jan Eickhoff, Christian Ottmann

**Affiliations:** † Lead Discovery Center GmbH, Otto-Hahn-Str. 15, 44227 Dortmund, Germany; ‡ Laboratory of Chemical Biology, Department of Biomedical Engineering and Institute for Complex Molecular Systems, Eindhoven University of Technology, Den Dolech 2, 5612 AZ Eindhoven, The Netherlands; § Taros Chemicals GmbH & Co. KG, Emil-Figge-Straße 76a, 44227 Dortmund, Germany; ∥ Department of Biotechnology, Chemistry and Pharmacy, University of Siena, Via Aldo Moro 2, 53100 Siena, Italy; ⊥ CNRS EMR9002 Integrative Structural Biology, University of Lille, F-59000 Lille, France; # University of Lille, Inserm, Institut Pasteur de Lille, U1167—RID-AGE—Risk Factors and Molecular Determinants of Aging-Related Diseases, F-59000 Lille, France

## Abstract

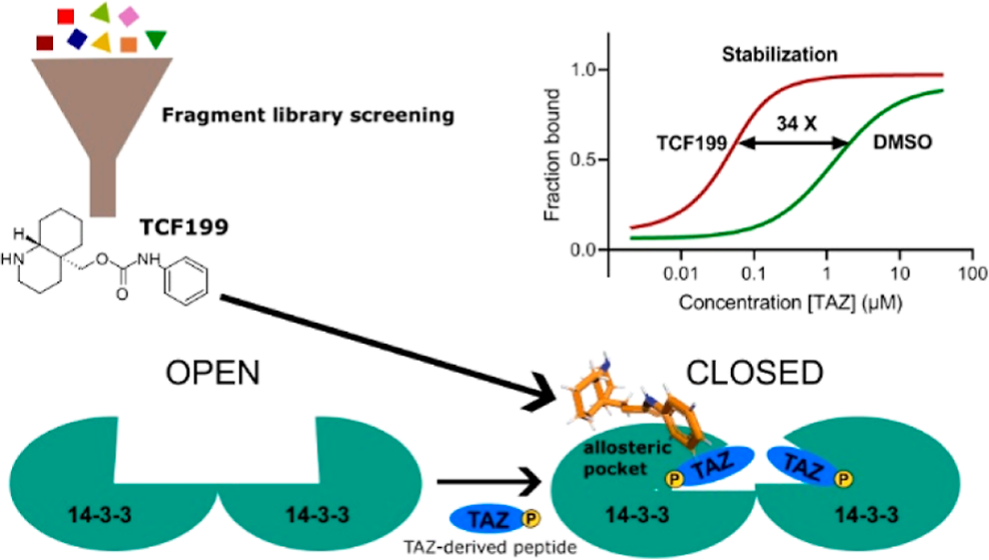

The identification of chemical starting points for the development of molecular glues is challenging. Here, we employed fragment screening and identified an allosteric stabilizer of the complex between 14–3–3 and a TAZ-derived peptide. The fragment binds preferentially to the 14–3–3/TAZ peptide complex and shows moderate stabilization in differential scanning fluorimetry and microscale thermophoresis. The binding site of the fragment was predicted by molecular dynamics calculations to be distant from the 14–3–3/TAZ peptide interface, located between helices 8 and 9 of the 14–3–3 protein. This site was confirmed by nuclear magnetic resonance and X-ray protein crystallography, revealing the first example of an allosteric stabilizer for 14–3–3 protein–protein interactions.

## Introduction

Fragment-based drug discovery (FBDD) has developed into an established approach in the drug discovery process that is complementary to traditional high-throughput screening campaigns. Unlike high-throughput screening where large chemical libraries containing 10^5–6^ compounds are utilized, a fragment library of a few hundred or a few thousand fragments can sample a large chemical space efficiently.^1^ The success of FBDD is illustrated by 7 FDA approved drugs, namely, vemurafenib,^2^ venetoclax,^3^ erdafitinib,^4^ pexidartinib,^5^ asciminib,^6^ sotorasib,^7^ and very recent capivasertib,^8^ an Akt inhibitor which was in combination with fulvestrant approved for treatment of breast cancer. Targeting protein–protein interactions (PPIs) is both a tremendously attractive and also significantly challenging approach in drug discovery.^9^ While in contrast to PPI inhibition, targeted stabilization of PPIs has been largely overlooked for some time,^10^ the recent extensive activities and successes of molecular glues^11,12^ and heterobifunctional degraders^13^ drug discovery have moved this modality to the forefront in the past few years.

14–3–3 proteins are a class of adaptor proteins that exert their biological function by recognizing linear phosphoserine/phosphothreonine motifs in several hundred partner proteins, which are modulated in their behavior upon binding to 14–3–3.^14−19^ The effect that 14–3–3 binding has is client-dependent and client-specific. For example, 14–3–3 binding leads to inhibition of kinases like CRaf^20,21^ and LRRK2^22,23^ but to activation of the tumor suppressor p53^24,25^ or the cystic fibrosis-related chloride channel CFTR.^26,27^ In many cases, 14–3–3 binding leads to functional inhibition by competing with other partner proteins, as is the case with Gab2^28,29^ and SOS1.^30,31^ Cytoplasmic sequestration of transcription factors is another important mechanism of regulation by 14–3–3, as in the case of FOXO1^32,33^ and ChREBP.^34,35^ The importance of 14–3–3 regulation is illustrated by clinically manifested mutations that abrogate or weaken 14–3–3 binding to clients like USP8^36,37^ or Pyrin^38,39^ leading to diseases like Cushing’s and Familial Mediterranean Fever, respectively.

In many cases, including the examples above, stabilization of 14–3–3 binding to a client can be expected to convey a therapeutic benefit. This has sparked interest in molecules like the diterpene glycoside Fusicoccin A, which has initially been identified as a stabilizer of 14–3–3 binding to a proton pump in plants,^40^ but together with close relatives and semisynthetic derivatives (fusicoccanes) have been shown to have multiple useful activities in human cells too.^19,41,42^ The considerable chemical complexity of fusicoccanes makes drug discovery efforts very challenging, which calls for alternative, more tractable chemistry to stabilize 14–3–3 PPIs. Fortunately, the interface between 14–3–3 and client proteins is amenable for both covalent^43−46^ and noncovalent^47−49^ fragment-based approaches.

Here, we focused on targeting the 14–3–3/TAZ interaction with fragments, which is a physiologically relevant PPI within the Hippo pathway. In recent years, the Hippo signaling pathway has come into the spotlight as a target for drug therapy, particularly due to its involvement in several pathologies, including cancer.^50,51^ When activated, the Hippo pathway acts as a tumor suppressor pathway in which TAZ is phosphorylated by upstream kinases and its activity is regulated by binding to 14–3–3, preventing TAZ translocation into the nucleus where it interacts with transcription factors such as TEADs and induces the transcription of genes associated with cancer.^52^ Stabilizing the 14–3–3/TAZ interaction would therefore be an attractive strategy for targeting cancers with aberrant Hippo pathway signaling. We report an allosteric fragment stabilizer of the interaction between 14–3–3 and the TAZ-derived peptide and have confirmed its mode of action by bioinformatics and biophysical methods. To the best of our knowledge, this is the first example of a functional stabilizing fragment of a 14–3–3 PPI that operates by an allosteric mechanism.

## Materials and Methods

### Differential Scanning Fluorimetry

Temperature-induced 14–3–3ζ unfolding was measured using the fluorescent dye GloMelt (Biotium; San Francisco, CA, USA). For testing fragments, 15 μL of assay mixture containing 1 μM 14–3–3ζ, 5 μM TAZpS89 peptide (QHVRSHpSSPASLQ; Caslo, Denmark), and 0.05% Tween20 and 1X GloMelt dye in DPBS (PAN-Biotech, Germany) was added to wells in a 384-well plate (Corning, Corning, NY, USA). Fragments were transferred to wells with the mixture using an acoustic liquid handler Echo 520 (Labcyte Inc., Sunnyvale, CA, USA). After 1 h of incubation, 10 μL of mixture was manually transferred to a 96-well PCR plate (Applied Biosystems, Waltham, MA, US) and spun down. Thermal denaturation-induced fluorescence was recorded on a PCR thermocycler StepOnePlus Real-Time PCR System (Applied Biosystems, Waltham, MA, USA). The protein melt profile consisted of an initial step at 25 °C for 30 s and a step where the plate was heated from 25 to 99 °C at a ramp rate of 0.05 °C/s. SYBR Green channel was used for readings of each heating step. Thermal denaturation data were analyzed with DMAN.^53^ Differential scanning fluorimetry (DSF) with the 14–3–3ζ/ERα-derived peptide (GGASVEETDQSHLATAGSTSSHSLQKYYITGEAEGFPApTV; Caslo, Denmark) and Fusicoccin A (Enzo Life Sciences, Inc., Farmingdale, NY, USA), and TAZpS89 peptide titration were performed in a similar manner as described above.

### Microscale Thermophoresis

14–3–3ζ was labeled using the Monolith Protein Labeling Kit RED-NHS according to the manufacturer’s protocol (NanoTemper Technologies, Germany). Briefly, 100 μL of 20 μM 14–3–3ζ was mixed with 100 μL of 66 μM NT647-NHS dye. The reaction was carried out in DPBS (PAN-Biotech, Germany) for 30 min in the dark at room temperature. After the reaction, the unbound dye was removed with the included gravity column. Labeled 14–3–3ζ was eluted with 600 μL of DPBS with 0.05% Tween20 aliquoted and flash-frozen. For fragment testing, 20 μL of assay mixture containing 15 nM labeled 14–3–3ζ, 150 nM TAZpS89 peptide, and 0.05% Tween20 in DPBS was added to wells in a 384-well plate (Corning, Corning, NY, USA). Fragments were transferred to wells with the mixture using an acoustic liquid handler Echo 520. After 1 h of incubation, Monolith Premium capillaries (NanoTemper Technologies, Germany) were loaded with the mixture and placed into the Monolith NT.115 (NanoTemper Technologies, Germany). Thermophoresis was read using a RED detector (Ex/Em: 650/670 nm) at 25 °C with settings at LED power of 25% and microscale thermophoresis (MST) power of 90%. Default settings were as follows: Fluo.Before 5 s, MST On 30 s, Fluo. After 5 s, the delay was 25 s. Data were analyzed with MO.Affinity Analysis Software (NanoTemper Technologies, Germany). For apparent *K*_d_, the TAZpS89 peptide was titrated against a fixed concentration of labeled 14–3–3ζ in the presence and absence of 1 mM fragment. For dose–response curves for TCF199 binding to the 14–3–3ζ/ERα peptide and 14–3–3ζ/Chibby peptide (KKTPPRKSApSLSNLHSLDRSTREVELGLEYGSPTMNLAGQ; Caslo, Denmark), 15 nM labeled 14–3–3ζ and 1.25 μM peptides were used. Fragments that induced a change in fluorescence were tested in the SD-test and fluorescence was plotted against the fragment concentration.

### Protein Expression and Purification

14–3–3ζ and 14–3–3σΔC (C-terminally truncated after T231) were expressed in a similar manner in BL21 (DE3) competent cells via a pProEX HTb plasmid. Expression was induced with 0.4 mM isopropyl β-d-1-thiogalactopyranoside (IPTG) overnight at 18 °C. After spinning down and lysis of the expression culture, the protein was purified on a Ni^2+^ –NTA column. The His6-tag in 14–3–3σΔC was cleaved with tobacco etch virus protease in a 1:0.05 mg ratio and a second nickel-affinity chromatography was performed followed by size exclusion chromatography (Superdex 75) in 25 mM Hepes pH = 7.5, 100 mM NaCl, 10 mM MgCl_2_, and 2 mM β-mercaptoethanol.

### Protein Crystallization and Structure Elucidation

Crystals of the 14–3–3σΔC/peptide complexes were grown by mixing 14–3–3σΔC (protein concentration, 12.5 mg·mL^–1^) in a molar ratio of 1:2 with the peptide, in 20 mM Hepes pH = 7.5, 2 mM MgCl_2_, and 2 mM β-mercaptoethanol, and incubated overnight at 4 °C. The complex was then set up for crystallization trials using the sitting drop method by mixing each complex 1:1 with different precipitation buffers. The precipitation buffers contained 0.095 M Hepes, PEG 400, 0.19 M CaCl_2_, and 5% glycerol, pH = 7.5, and PEG 400 26%. Crystals were grown within 7 days and could be directly flash-frozen in liquid nitrogen. For the soaking experiments, 100 mM stock solutions in DMSO of the compounds were added to the crystals to a final concentration of 10 mM. Crystals were harvested after 11–13 days of incubation and directly flash-cooled in liquid nitrogen. Data collection was performed with ESRF Beamline ID23–1. Data were processed using DIALS. Molecular replacement was carried out using a Phaser. The obtained model was subjected to reiterative rounds of model building and refinement by using Coot and Phenix. Figures were prepared by using PyMOL software.

### Molecular Dynamics Simulations

The 14–3–3ζ/TAZps89 complex was generated by structural superimposition using the crystallographic structure of the 14–3–3σ/TAZpS89 complex (PDB ID: 5N5W)^47^ as a structural template. Eight TCF199 molecules were arbitrarily and manually placed around the 14–3–3/TAZpS89 complex with a random orientation and at a distance higher than 30 Å from the protein complex. Then, TIP3P-type water molecules were added to solvate the simulation system in a rectilinear box having 5 Å of buffer from the solute. The total charge of the system was neutralized by Na^+^ ions. TCF199 was parametrized with the general Amber force field,^54^ while the protein was parametrized with the ff14SB force field. Parameters for phosphorylated serine were retrieved from the AMBER Parameter Database.^55^ Molecular dynamics (MD) simulations were carried out with Amber18^56^ using the protocol described previously.^57−59^ Three independent MD replicas of 500 ns each were run. MD trajectories analysis was carried out with CPPTRAJ software.^60^ Cluster analysis of MD frames was achieved by using a hierarchical agglomerative (bottom-up) approach, and the representative frame of the most populated cluster was used for graphic representations.

### Nuclear Magnetic Resonance Experiments

Details of ^15^N^2^H isotope-labeled 14–3–3σΔC17 protein preparation and nuclear magnetic resonance (NMR) experiments were previously reported^61^ and are here briefly described. The same protocol was followed for the labeling of 14–3–3ζ. Backbone assignments of ^15^N^2^H-labeled 14–3–3σΔC17 were previously reported.^62^ Spectra were acquired here using a 900 MHz NEO cryogenic probe. The reference for the ^1^H chemical shift was relative to trimethylsilyl propionate. The WaterLOGSY spectra were recorded at 289 K with 32k complex data points and with a mixing time of 1.7 s. The number of scans per increment was 640. NMR buffer contained 100 mM sodium phosphate, 30 mM NaCl, pH = 6.55, and 5% (v/v) D_2_O. The final concentration of DMSO-*d*_6_ was 5% in all samples. ^1^H–^15^N TROSY-HSQC spectra of 75 μM ^15^N^2^H-labeled 14–3–3σΔC17 were acquired with 3072 complex data points in the direct dimension and 120 complex data points in the indirect dimension, with 128 scans per increment at 305 K, in a buffer containing 100 mM sodium phosphate, 50 mM NaCl, pH = 6.8, 1 mM DTT, EDTA-free protease inhibitor cocktail (Roche, Basel, Switzerland), and 10% (v/v) D2O. The final concentration of DMSO-*d*_6_ was 2% and was kept constant for all the experiments. Spectra were collected and processed with Topspin 4 (Bruker Biospin, Karlsruhe, Germany). ^1^H, ^15^N combined chemical shift value modifications were calculated as Δδ = (Δδ(^1^H)2 + 0.12*Δδ(^15^N)2)1/2 where Δδ is the difference (Δ) in chemical shift value (δ) between the bound and free states of the protein on the proton scale Δδ(^1^H) or ^15^N scale Δδ ^15^N, the latter adjusted for the larger dispersion on this dimension (*0.12).

### Statistical Analysis

Graphs were fitted with GraphPad Prism 9.0. For MST analysis, fragment concentrations that generated aberrant MST traces were dismissed as well as obvious outliers.

## Results and Discussion

### Assay Setup to Identify Molecules Binding to the 14–3–3/TAZ Complex

**Figure 1:**
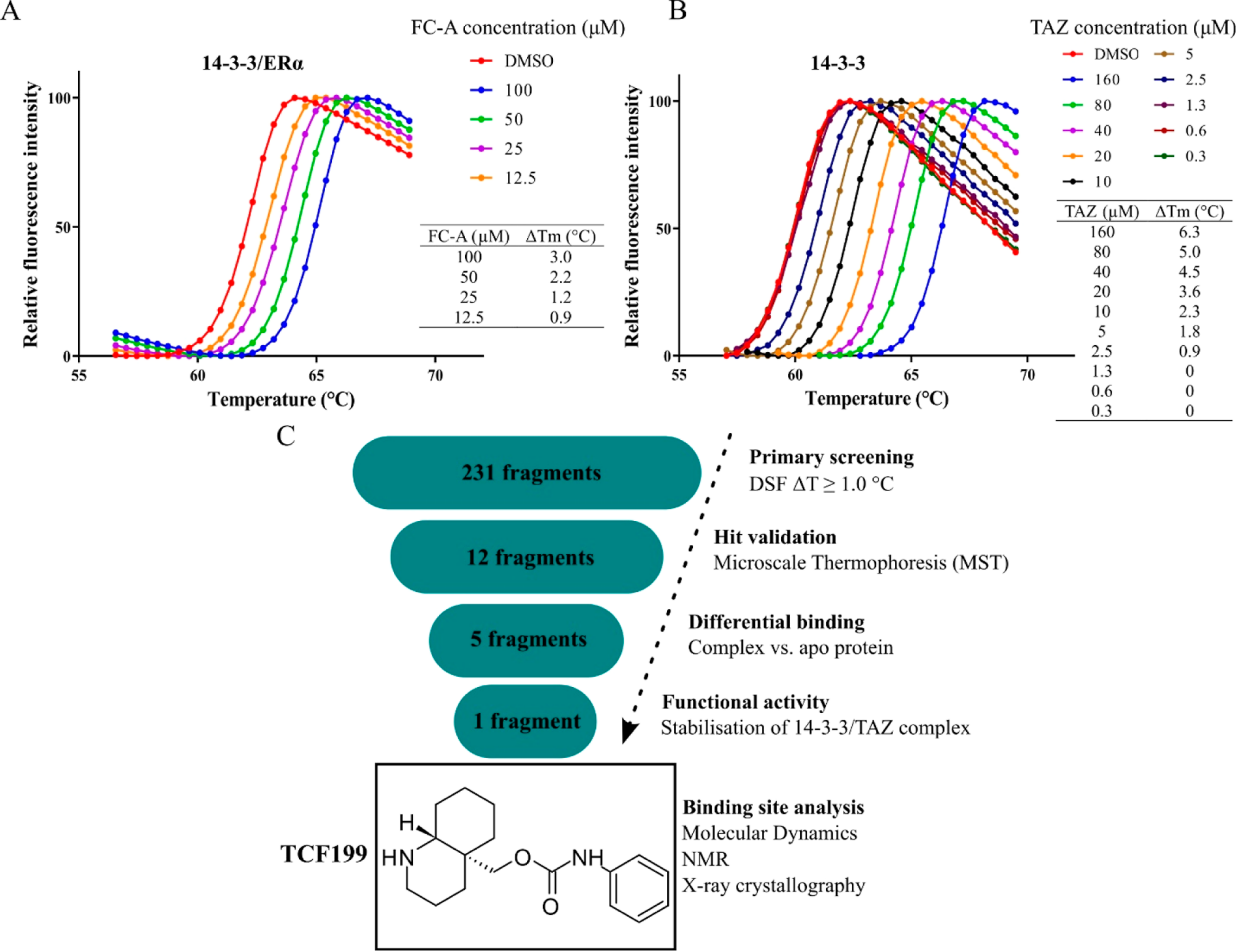
Assay setup and screening cascade. (A) Thermal denaturation curves of Fusicoccin A (FC-A) binding to the 14–3–3/ERα binary complex. Plotted are mean values of triplicates. The table shows the *T*_m_ shift (Δ*T*_m_) for the indicated FC-A concentration. (B) Thermal denaturation curves upon binding of TAZ to 14–3–3. Table displays *T*_m_ shift (Δ*T*_m_) for the indicated TAZ concentration. (C) Screening cascade started with the DSF as a primary screen. Next, screening hits were tested by MST to confirm binding to the 14–3–3/TAZ complex. Confirmed hits were further tested for differential binding to apo 14–3–3. Fragment TCF199 showed preferential binding to the binary 14–3–3/TAZ complex. Next, TCF199 was tested for its ability to stabilize the 14–3–3/TAZ complex, and finally, the ternary complex 14–3–3/TAZ/TCF199 was characterized by MD, NMR spectroscopy, and X-ray crystallography.

**Figure 2:**
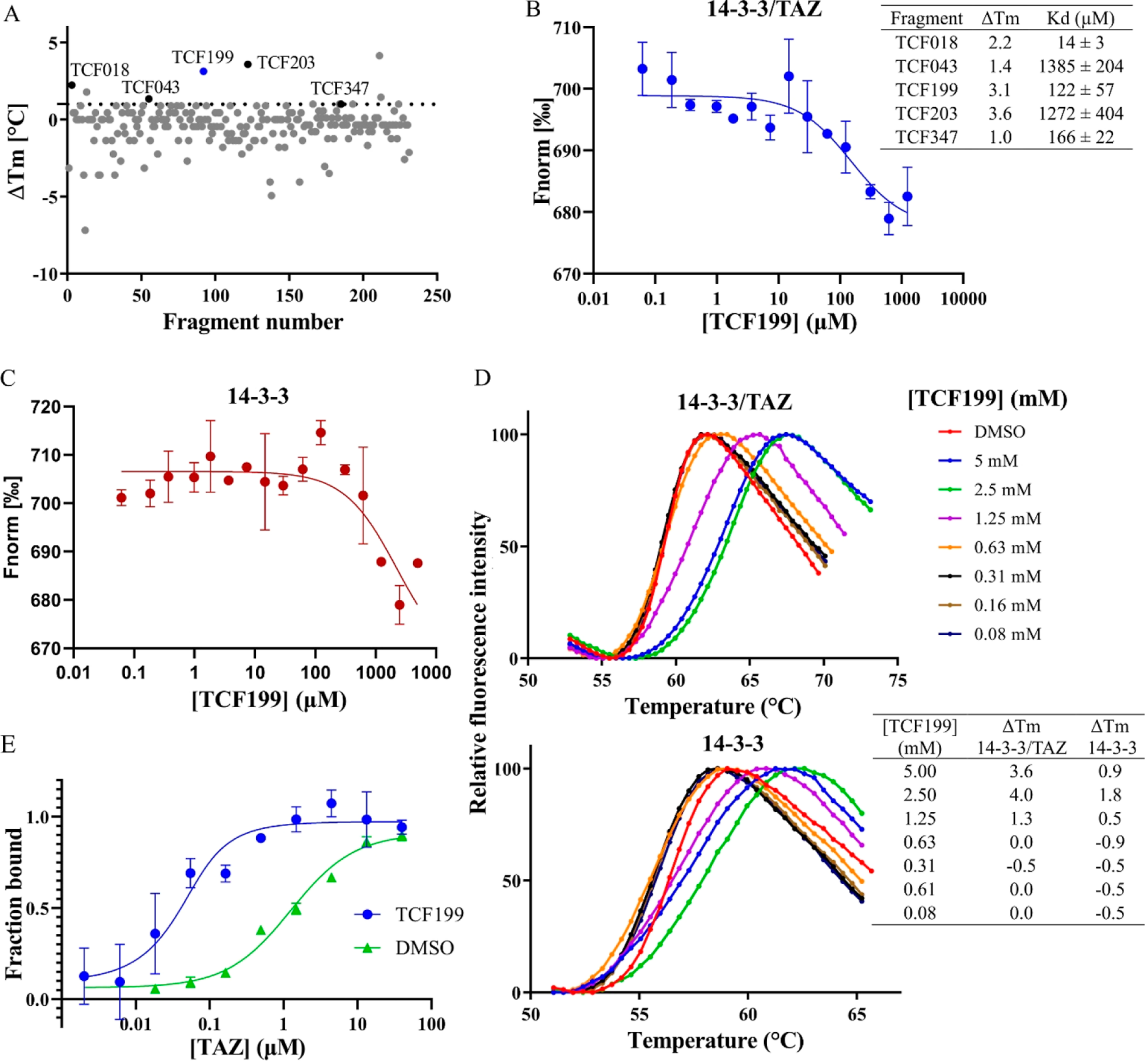
Fragment screen and hit validation. (A) Fragment screening results. On the *y* axis are the Δ*T*_m_ values generated by the fragments, calculated from the average *T*_m_ of the DMSO controls. The dotted line represents the hit selection criteria, which was set at Δ*T*_m_ ≥ 1.0 °C. Black circles represent primary hits that passed the DSF selection criterion and were verified by MST for binding to the 14–3–3/TAZ complex. The blue circle depicts fragment TCF199. Hits that did not show a clear transition from native to the denatured state are not plotted. (B) Dose–response curve of TCF199 binding to the 14–3–3/TAZ complex (mean ± SD; *n* = 2) measured by MST. The table shows the comparison of *K*_d_ and Δ*T*m values from (A) of the indicated fragments. (C) Dose–response curve of TCF199 binding to apo 14–3–3 (mean ± SD; *n* = 2; *K*_d_ = > 2 mM) measured by MST. (D) Thermal denaturation curves of the 14–3–3/TAZ complex (top) and apo 14–3–3 (bottom) incubated with indicated concentrations of TCF199 for 1 h prior to DSF (mean, *n* = 3). The table shows the comparison of Δ*T*_m_ between 14–3–3/TAZ complex and apo 14–3–3 for indicated concentrations of TCF199. (E) Apparent *K*_d_ of TAZ binding to 14–3–3 in the presence (blue; *K*_d_ = 0.04 ± 0.02 μM) and absence (green; *K*_d_ = 1.38 ± 0.44 μM) of 1 mM TCF199 (mean ± SD, *n* = 3) measured by MST.

Differential scanning fluorimetry (DSF) is a well-established biophysical technique for fragment screening. It detects the temperature-dependent unfolding of a protein by binding of a fluorescent dye to the unfolded state of a protein. Upon binding of a ligand to a protein, a shift in the melting temperature is observed (Δ*T*_m_ = *T*_m_ (protein + ligand) – *T*_m_ protein). Compared to other fragment screening techniques such as X-ray crystallography, NMR, and surface plasmon resonance, DSF is a simple and rapid technique that does not require dedicated equipment. Fragments tend to be weak binders and are typically screened at millimolar (mM) concentrations, making them more susceptible to aggregation and interference; therefore, the addition of detergents to a screening buffer is recommended. For this reason, we used fluorescent dye GloMelt in our assay, which does not interfere with the readout in the presence of detergents. Routinely, DSF is employed to detect binary interactions; however, we first determined whether DSF is suitable for our assay to distinguish compounds that bind to a binary complex, as we wanted to screen the fragment library against the 14–3–3/TAZ complex. Fusicoccin A serves as a robust positive control that binds to the 14–3–3/estrogen receptor alpha (ERα) complex and stabilizes the interaction.^63^ In order to validate the assay, we incubated FC-A with the 14–3–3/ERα-derived peptide for 15 min and subsequently performed DSF. Indeed, we observed a dose-dependent increase in melting temperature (Figure 1A), confirming that the assay is able to identify binders of a binary complex. Prior to the screening of a fragment library, we first verified that we could detect an increase in melting temperature upon binding of the TAZ-derived peptide to 14–3–3, forming a binary complex. Upon binding of TAZ to 14–3–3, a dose-dependent shift in melting temperature was observed, indicative of binary complex formation (Figure 1B). With this assay in hand, we screened the fragment library against the 14–3–3/TAZ complex (Figure 1C). Primary screen hits were tested by MST to validate binding to 14–3–3/TAZ. Validated hits were further investigated for differential binding to apo 14–3–3. Fragment TCF199 showed a higher affinity for the 14–3–3/TAZ complex compared to apo 14–3–3. Although the fragments are small and generally do not show functional activity on the protein, we performed MST to determine the apparent *K*_d_ of the TAZ peptide to 14–3–3 in the presence and absence of TCF199 (Figure 2). However, gratifyingly, the fragment stabilized the 14–3–3/TAZ peptide interaction. The binding of this fragment was further characterized by MD, NMR spectroscopy, and X-ray crystallography.

### Fragment Library Screen and Hit Validation

As mentioned above, the screening cascade was initiated with the primary screening of 231 fragments against the 14–3–3/TAZ complex. Fragments were screened as singletons at a one-point concentration of 5 or 2.5 mM, depending on the solubility of the fragments in DMSO. Each assay plate contained DMSO controls, which were used to calculate Δ*T*_m_ for wells containing fragments [Δ*T*_m_ = *T*_m_ (complex + fragment) – average *T*_m_ (complex + DMSO)]. The hit selection criterion was set at Δ*T*_m_ ≥ 1.0 °C. Fragments with a negative *T*_m_ shift were dismissed since they preferentially bind to denatured or partially denatured parts of a protein.^64^ Fragments generating a poorly resolved transition from the native to unfolded state were dismissed. After applying these filters to the screening results, we obtained 12 hits (Figure 2A). We followed up with these hits using MST as an orthogonal assay. The hits were tested in 16-point dose response curves to validate binding to the 14–3–3/TAZ complex. Five hits were confirmed to bind a binary complex with *K*_d_ values ranging from 2-digit micromolar for TCF018, high micromolar for TCF199 and TCF347, and up to millimolar for TCF043 and TCF203 (Figures 2B and S1). Interestingly, the shifts in melting temperatures do not correlate with the *K*_d_ values obtained with the MST, suggesting that DSF only provides the information on whether the fragment is binding or not; however, a trend of increasing *T*_m_ with an increasing fragment concentration can still be observed.

### Fragment TCF199 Stabilizes the 14–3–3/TAZ Interaction

Although the aim of the fragment screening was to discover new fragments that bind to the complex or to 14–3–3 alone and could potentially be used as a starting point for the development of compounds that could stabilize the complex, we further investigated validated hits for differential binding to 14–3–3 alone. Differential binding of a fragment to apo 14–3–3 would indicate whether a fragment interacts with both partners (14–3–3 and TAZ-derived peptide) simultaneously (orthosteric PPI stabilizer) or a fragment binds elsewhere on 14–3–3 and favors the TAZ bound state more indirectly (allosteric PPI stabilizer). Interestingly, TCF199 showed a significantly lower affinity for the apo 14–3–3 (*K*_d_ = > 2 mM) compared to the 14–3–3/TAZ complex (compare Figure 2C and Figure 2B; Figure S2). In order to confirm this behavior of the fragment, we performed DSF with TCF199 incubated with 14–3–3/TAZ or 14–3–3 alone, and indeed, a similar behavior was observed (Figure 2D). Similarly, we have observed a modification of the signal intensity in 1D NMR WaterLOGSY experiments when 14–3–3σ/TAZ or 14–3–3ζ/TAZ was added to TCF199, compared to the addition of apo 14–3–3 (Figure S3A,B), again strongly suggesting differential binding to the complex for both isoforms. With this in mind, we next tested this fragment for its ability to stabilize the 14–3–3/TAZ interaction. We performed MST to measure the apparent *K*_d_ for the TAZ peptide to 14–3–3 in the presence and absence of TCF199. Based on the earlier obtained *K*_d_ values for the fragment, we selected a concentration of 1 mM to achieve full saturation and titrated the TAZ peptide against 14–3–3. To our positive surprise, fragment TCF199 showed a stabilization effect with a 34-fold change in the *K*_d_ value of TAZ binding to 14–3–3, from 1.38 ± 0.44 μM to 0.04 ± 0.02 μM (Figure 2E). TCF199 was one of the fragments added to the fragment library as a collection of fragments inspired by natural products. The chemical synthesis of compounds with such scaffolds has been described elsewhere.^65^

### Binding Site Analysis

**Figure 3:**
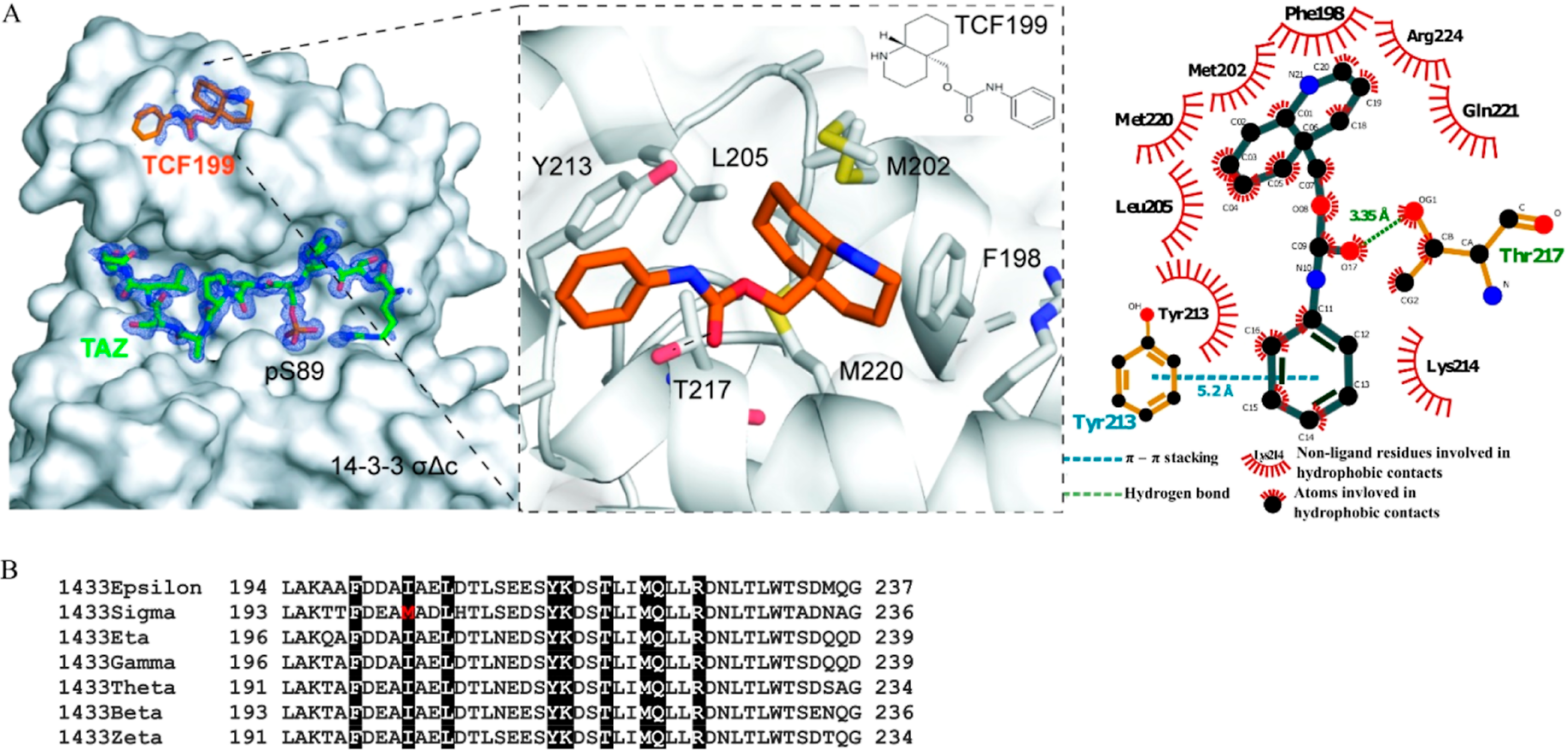
Binding site analysis. (A) High-resolution crystal structures of fragment TCF199 (orange sticks) in complex with 14–3–3σΔC protein (white surface) and the TAZpS89 peptide (green sticks) (PDB ID: 8R0Z). Detailed overview of TCF199 binding site (middle). 2D plot of 14–3–3 and TCF199 interactions (right-hand side). 2D plot was created with LigPlot+. (B) Alignment of selected amino acid sequences of human 14–3–3 isoforms. Amino acids that come into contact with TCF199 are highlighted.

Next, we sought to identify the binding site of fragment TCF199 in the 14–3–3/TAZ complex and characterize its binding. The crystal structure of TAZpS89 bound to 14–3–3σ has been elucidated previously.^47^ Briefly, the TAZ-derived peptide surrounding phosphorylated Ser89 (pS89) completely occupies the 14–3–3’s binding groove (Figure S3B,C). Prior to the soaking of TCF199 to crystals of the 14–3–3/TAZpS89 complex, we performed MD simulations in order to identify potential binding sites and binding modes of TCF199 in the 14–3–3ζ/TAZ complex. To this aim, three replicas of 500 ns unbiased MD simulations were run for a total simulation time of 1.5 μs, starting from an unbounded state in which multiple units of TCF199 were placed in the solvent box with a random orientation and at a distance higher than 30 Å from the 14–3–3/TAZpS89 complex. Statistically significant results hinted that the bound TCF199 is located on a site at the upper edge of the 14–3–3’s binding groove and is therefore not interacting directly with the TAZ peptide (Figure S4). Moreover, two possible orientations of TCF199 within the site were identified by MD simulations (Figure S4B,C). We used the results of MD analysis as a guideline for determining the crystal structure of the 14–3–3σΔC/TAZpS89/TCF199 complex. In agreement with MD results, additional electron density from measuring crystals soaked with the fragment could be observed in a pocket distant to the peptide binding site, between helix 8 and 9 of 14–3–3 (Figures 3A and S5). Although electron density coverage was not complete for the entire molecule, we could build the fragment in a possible orientation where it sits in a hydrophobic pocket with amino acids Phe198, Met202, Leu205, Thr217, Met220, Gln221, and Arg224 interacting with the fragment’s fused ring structure (decahydroquinoline). The carbonyl group interacts via a hydrogen bond with Thr217, and the phenyl ring interacts with Tyr213 via Y-shaped π – π stacking and via hydrophobic interaction with Lys214. The phenyl ring shows a lower electron density, suggesting higher flexibility or the presence of different conformations. The binding mode observed via X-ray crystallography is comparable to that investigated by MD simulations (Figure S6) especially for the interaction of the decahydroquinoline moiety and the carbonyl ring, whereas a deviation on the position of the phenyl ring is observed. However, this is coherent with the lower electron density and the possibility for this ring to occupy multiple sites at the 14–3–3/TAZ interface. 2D NMR spectra of 14–3–3σ complexed with the TAZ-derived peptide or in the apo form together with the TCF199 compound (Figure S7) showed modest chemical shift value modifications affecting a few resonances. Among these, the resonance of Thr217 showed a chemical shift value deviation, in the presence of both apo 14–3–3 and 14–3–3σ/TAZ, confirming the pocket identification. The similarity of the perturbations observed in the spectra of the apo and complexed forms indicated that the TCF199 binding mode was independent of the presence of the peptide. The hydrophobic pocket where TCF199 binds has been identified as a target for fragments in at least two other screens,^47,61^ however, the discovered fragments did not show any stabilizing effect on the interaction between 14–3–3 and a client protein/peptide. The pocket where the fragment binds is conserved across 14–3–3 isoforms with a difference for the σ isoform, which has a methionine at position 202, whereas other isoforms display an isoleucine (Figure 3B).

### TCF199 Locks 14–3–3 in a Closed Conformation

**Figure 4:**
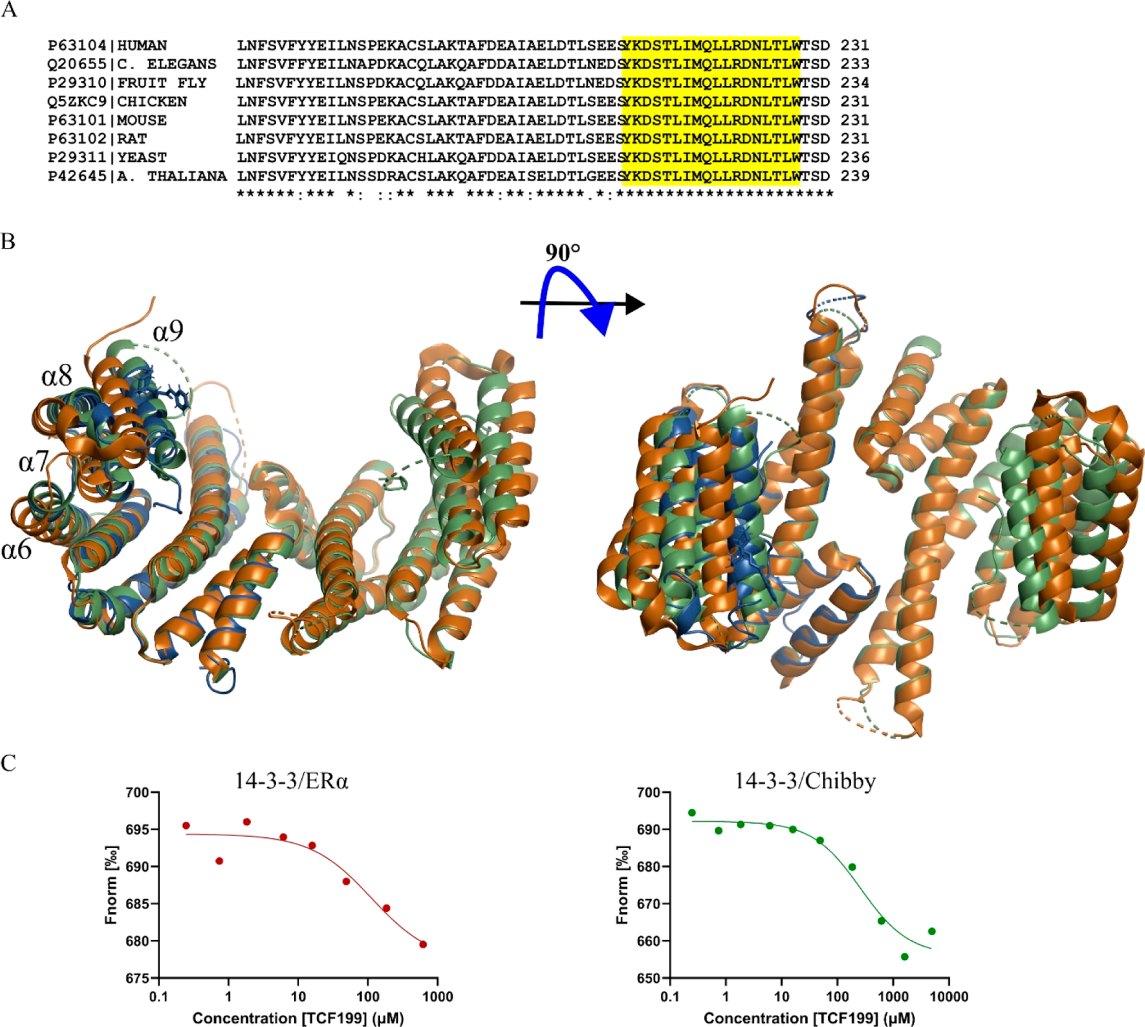
TCF199 holds 14–3–3 in closed conformation. (A) Alignment of selected amino acid sequences of 14–3–3 proteins of different eucaryotes. Amino acids comprising helix 9 are highlighted. (B) Superimposition of 14–3–3σ in open conformation (PDB ID: 5OMA; orange), closed conformation (PDB ID: 5OKF; olive), and the crystal structure reported here (PDB ID: 8R0Z; blue). (C) Dose–response curves of TCF199 binding to 14–3–3/ERα (*K*_d_ = 112 ± 77 μM) and 14–3–3/Chibby (*K*_d_ = 260 ± 102 μM) complexes.

14–3–3s are considered to be rigid proteins that act as molecular anvils for partner proteins, facilitating conformational changes of their binding partners.^66^ While this is largely correct, several studies have also shown that 14–3–3s display some flexibility and transit between “open” and “closed” conformations. Comparison of apo 14–3–3 and 14–3–3 in complex with the client peptide/protein, revealed that the conformation depends on whether the ligand is bound (closed conformation) or not (open conformation).^67−73^ The highest degree of flexibility was shown for helix 9, which is notably conserved among eukaryotes (Figure 4A), suggesting that this may be a general feature of all 14–3–3s. Furthermore, it was shown that the observed conformational change is not caused by crystal packing, as these dynamic transitions were also detected in solution.^69^ We compared our 14–3–3/TAZ/TCF199 crystal structure with the closed and open 14–3–3 conformations (PDB ID: 5OKF and 5OMA).^71,73^ Superimposition of the crystal structures indicates that our structure is similar to the crystal structure of 14–3–3 in the closed conformation (5OKF), where helices 6–9 are in a different position compared to 14–3–3 in the open conformation (5OMA) (Figure 4B). Based on the data obtained here, we speculate that TCF199 binds to TAZpS89-bound 14–3–3, which has a closed conformation and links helices 8 and 9 together. This results in a reduced flexibility of helices 8 and 9. Therefore, bound TCF199 stabilizes 14–3–3 in a closed conformation and hinders TAZpS89 from leaving the binding groove.

The distance between fragment TCF199 and the TAZ peptide is substantial, with no direct interaction, hinting that it would be difficult to achieve selectivity for the client-derived peptides bound in the 14–3–3’s groove. Indeed, when we titrated TCF199 against 14–3–3 complexed with ERα or Chibby-derived peptides, we obtained similar *K*_d_ values to those for 14–3–3/TAZpS89 (Figure 4C). However, based on the deposited PDB structures of 14–3–3s complexed with full-length client proteins (Figure S8), it is evident that the upper edge of 14–3–3, where TCF199 is located, also participates in binding to client proteins (PDB IDs: 6U2H, 6GN8, 5LTW, 1IB1),^74−77^ suggesting that achieving selectivity for the client protein would be possible through additional interactions by elaboration of the fragment. Furthermore, helix 9 has been shown to be important for microtubule affinity regulating kinase 3 (MARK3) binding to 14–3–3, as well as in 14–3–3-mediated Ras-Raf signaling in *Drosophila*.^78,79^ Point mutation studies in 14–3–3 binding revealed that the exchange of tyrosine 213 (human isoform σ numbering) to phenylalanine impaired client protein binding, suggesting that binding partners, in addition to the phospho-binding groove, also make contacts with this region. Moreover, in a recent study by Petrvalska et al.,^80^ they characterized the 14–3–3/CaMKK1 binding by hydrogen/deuterium exchange coupled to MS (HDX–MS) which showed that 14–3–3’s helices 7–9 participate in CaMKK1 binding. Given the significance of the pocket to which TCF199 binds, it would be interesting to explore this pocket further in terms of chemical matter and activity or to use TCF199 as a starting point for medicinal chemistry efforts to selectively stabilize the interaction between 14–3–3 and full-length client proteins such as TAZ.

## Conclusions

FBDD is an established approach for generating high quality lead compounds, especially in the context of PPIs, which are considered hard-to-drug. Here, we implemented fragment screening against the 14–3–3/TAZpS89 complex, a physiologically relevant PPI within the Hippo pathway. The screen yielded a fragment inspired by natural products which bound to a conserved hydrophobic pocket located at the edge of the 14–3–3̀s binding groove. Although the fragment did not make direct contacts with TAZpS89, it stabilized the 14–3–3/TAZpS89 complex in an allosteric fashion. The stabilization effect might be explained through altering the 14–3–3’s flexibility of helices 8 and 9. We hypothesize that fragment TCF199 reduces the movement of helices 8 and 9 and therefore diminishes their flexibility, subsequently keeping 14–3–3 in closed conformation and hindering TAZ from leaving the binding groove. To the best of our knowledge, this is a first characterized allosteric binder of 14–3–3 that shows a stabilizing effect.

## Supporting Information Available

Hit validation, fragments binding to apo 14–3–3, characterization of TCF199 binding to 14–3–3 using NMR experiments, MD simulations, TCF199̀s binding site analysis, comparison MD vs X-ray structure, characterization of TCF199 binding to 14–3–3σΔC and 14–3–3σΔC/TAZ using NMR experiments, representative structures of near full-length proteins in complex with 14–3–3, data collection and refinement statistics, and method description for the SD-test (PDF)

## Accession Codes

14–3–3sigma, human: UNIPROT P31947·1433S_HUMAN, 14–3–3zeta, human: UNIPROTP63104·1433Z_HUMAN.
