# Supplementary material for: Fragment-Based Interrogation of the 14–3–3/TAZ Protein–Protein Interaction
Source: Biochemistry. 2024 Aug 22;63(17):2196–206. doi: 10.1021/acs.biochem.4c00248 (PMC11375770; doi:10.1021/acs.biochem.4c00248)
Supplement: Supplementary file 1 — bi4c00248_si_001.pdf [file bi4c00248_si_001.pdf]

Fragment-based interrogation of the 14-3-3/TAZ protein-protein interaction

Blaž Andlovic[ab], Dario Valenti[bc], Federica Centorrino[b], Francesca Picarazzi[d], Stanimira Hristeva[c], Malgorzata Hiltmann[a], Alexander Wolf[a], François-Xavier Cantrelle[ef], Mattia Mori[d], Isabelle Landrieu[ef]*, Laura M. Levy[c], Bert Klebl[a], Dimitrios Tzalis[c]*, Thorsten Genski[c], Jan Eickhoff[a]*, Christian Ottmann[b]**

[a] Lead Discovery Center GmbH, Otto-Hahn-Str. 15, 44227 Dortmund, Germany.

[b] Laboratory of Chemical Biology, Department of Biomedical Engineering and Institute for Complex Molecular Systems, Eindhoven University of Technology, Den Dolech 2, 5612 AZ Eindhoven, The Netherlands.

[c] Taros Chemicals GmbH & Co. KG, Emil-Figge-Straße 76a, 44227 Dortmund, Germany.

[d] Department of Biotechnology, Chemistry and Pharmacy, University of Siena, Via Aldo Moro 2, 53100 Siena, Italy.

[e] CNRS EMR9002 Integrative Structural Biology, F-59000 Lille, France. Univ. Lille.

[f] Univ. Lille, Inserm, Institut Pasteur de Lille, U1167 – RID-AGE - Risk Factors and Molecular Determinants of Aging-Related DiseasesF-59000 Lille, France.

*Emails: mattia.mori@unisi.it, isabelle.landrieu@univ-lille.fr, dtzalis@taros.de, eickhoff@lead-discovery.de, c.ottmann@tue.nl

Supporting Information

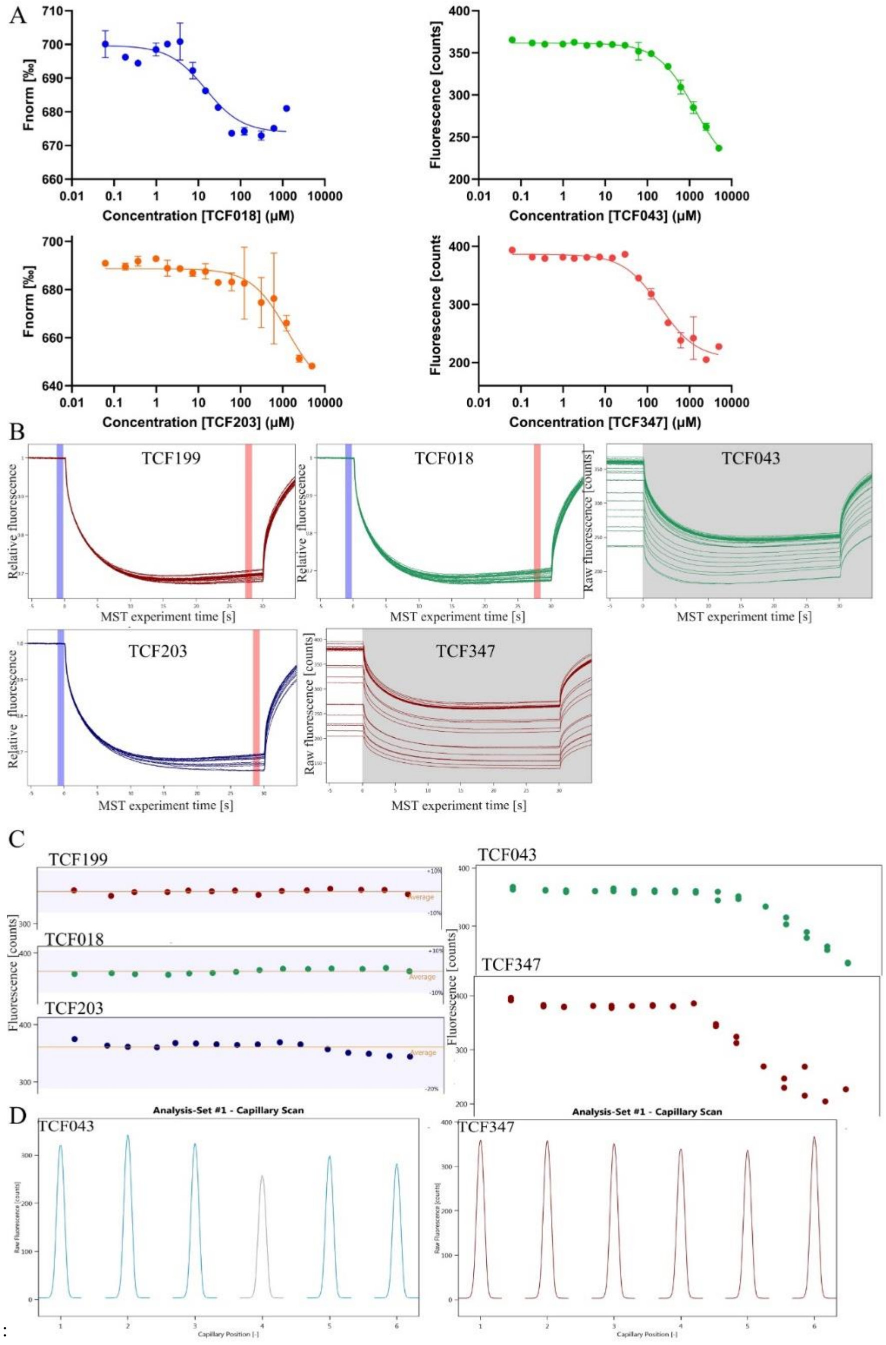

Figure S1: Hit validation (A) Dose-response curves of TCF018 (Kd = 14 ± 3 µM), TCF043 (Kd = 1385 ± 204 µM), TCF203 (Kd = 1272 ± 404 µM), and TCF347 (Kd = 166 ± 22 µM) binding to 14-3-3/TAZ complex (mean±SD; n=2). Analysis of TCF043 and TCF347 binding to 14-3-3/TAZ was carried out using fluorescence. (B) MST trace when IR-laser is off (blue line) and when the IR-laser is on (after blue line). Analysis was carried out using blue and red lines for fragments TCF199, TCF203 and TCF018. Fluorescence quenching is seen upon binding of TCF043 and TCF347. (C) Measurements of initial fluorescence at different fragments´ concentrations (concentration increases from left to right). Fragments TCF043 and TCF347 show fluorescence change upon binding. (D) Capillary scan after SD-test for fragments TCF043 and TCF347. Three highest and three lowest fragment concentrations were used for SD-test. For TCF043 a fourth capillary scan was excluded (grey).

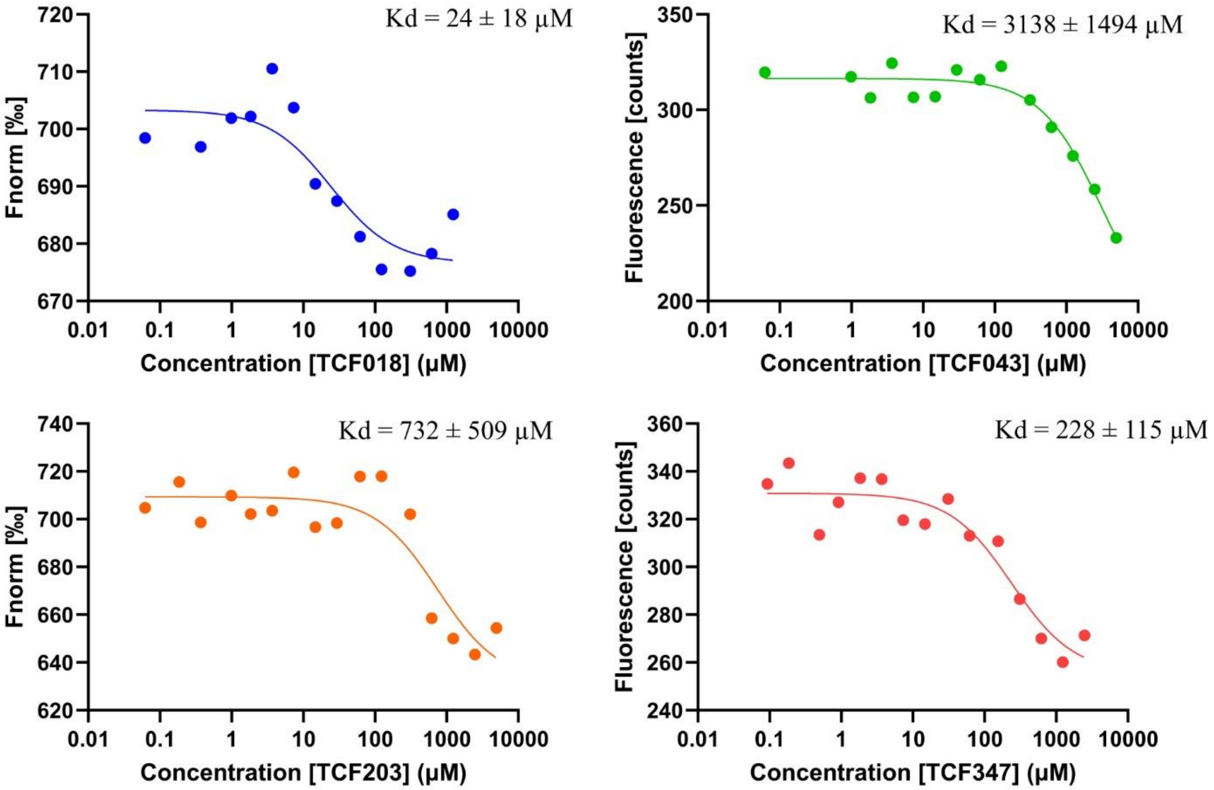

Figure S2: Fragments binding to apo 14-3-3. Dose-response curves of TCF018, TCF043, TCF203 and TCF347 binding to 14-3-3 alone. Analysis of TCF043 and TCF347 binding to apo 14-3-3 was carried out using fluorescence.

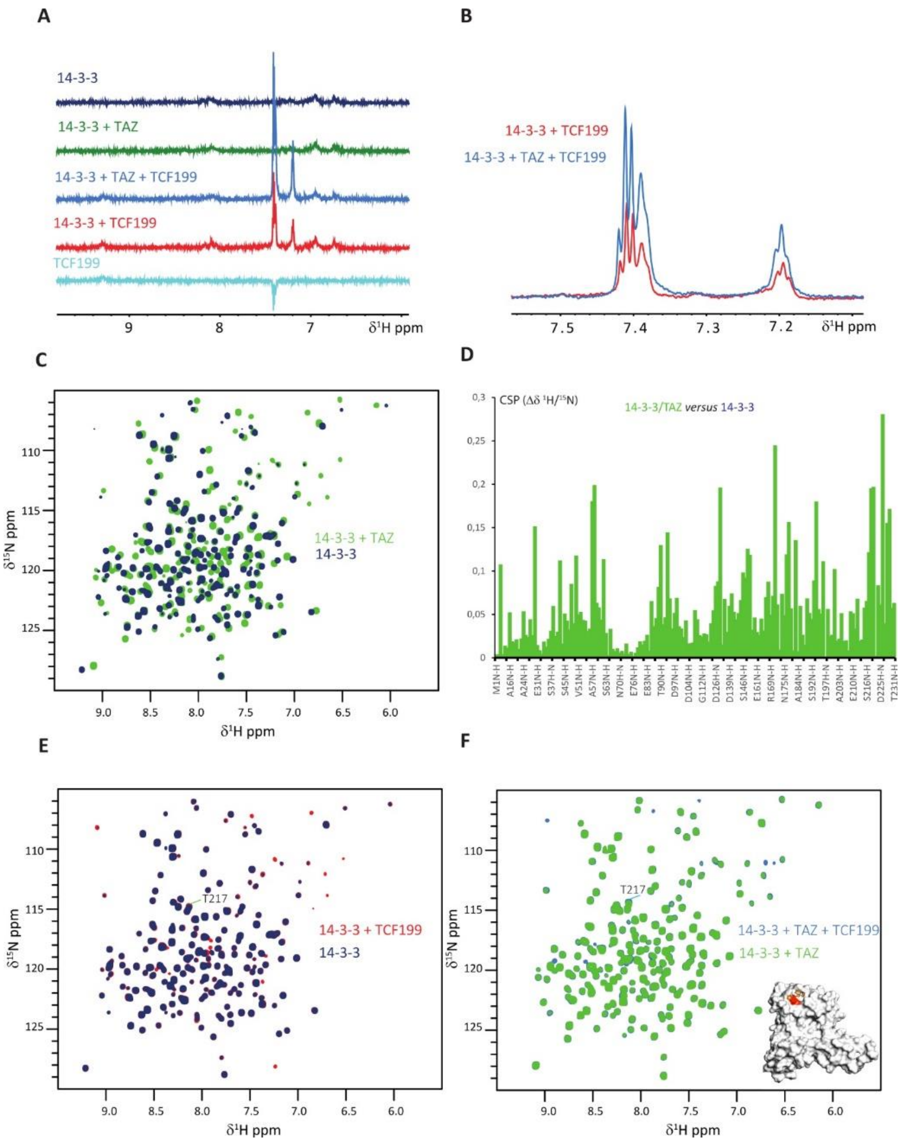

Figure S3: Characterization of TCF199 binding to 14-3-3σΔC and 14-3-3σΔC/TAZ using NMR experiments. (A) WaterLOGSY experiments corresponding to TCF199 (2 mM, negative control in light blue), 14-3-3σΔC in the apo form and 14-3-3σΔC complexed to TAZ (25 µM and 25µM/500µM, control experiments in dark blue and in green, respectively), apo-14-3-3σΔC in the presence of TCF199 (25µM/2 mM, in red) and 14-3-3σΔC in the presence of TAZ and of TCF199 (25µM/500µM/2 mM, in blue). The represented spectral region shows aromatic protons and signals detected from the TCF199 compound only (signals at 7.4 and 7.6 ppm not present in the 14-3-3σΔC and 14-3-3σΔC/TAZ control experiments). Binding to 14-3-3σΔC and 14-3-3σΔC/TAZ was evidenced as a sign inversion of these signals from negative values in TCF199 alone control experiment (in light blue) to positive values for both apo-14-3-3σΔC in the presence of TCF199 (in red) and 14-3-3σΔC in the presence of TAZ and of TCF199 (in blue). (B) The increased intensity of these signals in the Water LOGSY spectrum of 14-3-3σΔC/TAZ/TCF199 compared to the corresponding signals in 14-3-3σΔC/TCF199 suggested an higher affinity of TCF199 for the complex. (C) 2D ^{1}H-^{15}N TROSY-HSQC spectra of 75 µM ^{15}N^{2}H labeled 14-3-3σΔC in the presence of the TAZ peptide (in green) and in the absence (overlayed in dark blue). (D) Combined ^{1}H, ^{15}N chemical shift modifications between the spectra presented in (B), showing large chemical shift perturbations of multiple resonances following binding of the TAZ peptide. (E) 2D ^{1}H-^{15}N TROSY-HSQC spectra of 75 µM ^{15}N^{2}H labeled 14-3-3σΔC in the presence of TCF199 (in red) and in the absence (overlayed in dark blue) (F) 2D ^{1}H-^{15}N TROSY-HSQC spectra of 75 µM ^{15}N^{2}H labeled 14-3-3σΔC complexed to TAZ in the presence of TCF199 (in light blue) and in the absence (overlayed in green). (E-F) Resonance of H-^{15}N Thr217 that showed a small chemical shift value modification upon TCF199 binding is labelled.

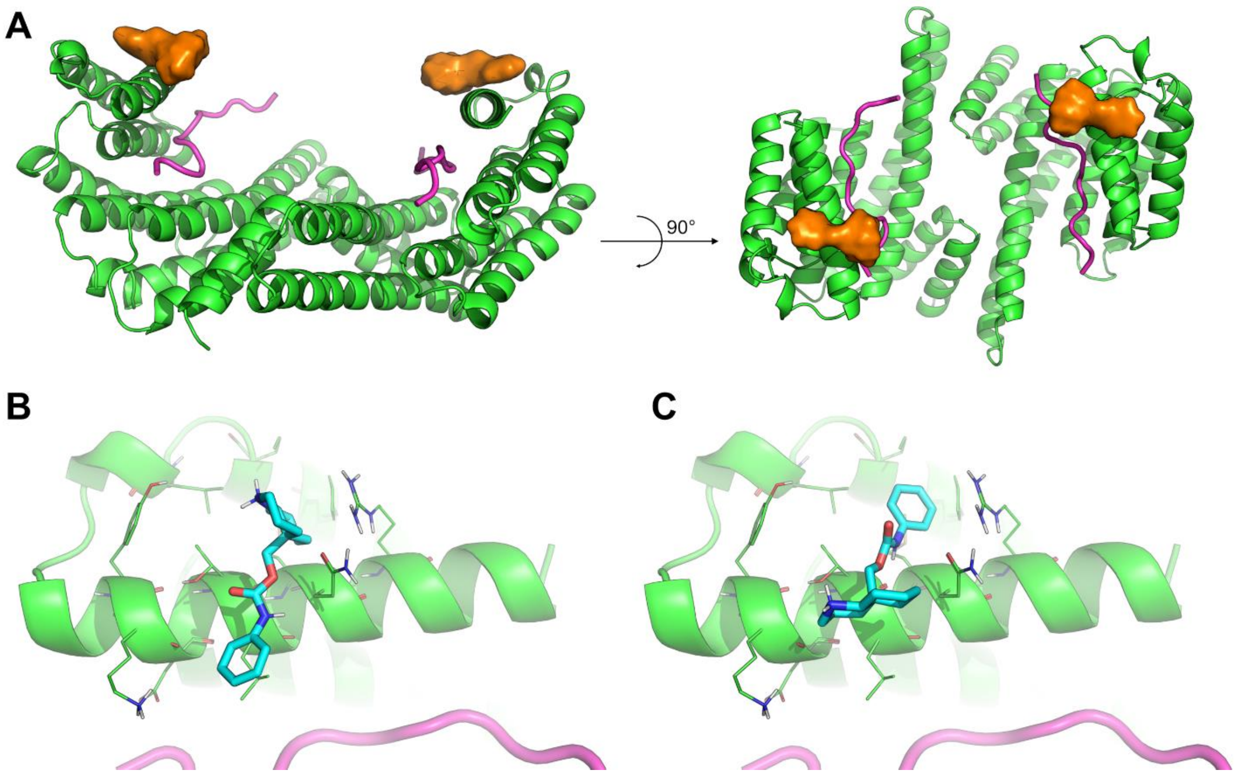

Figure S4: MD simulations. A) Statistically significant binding site of TCF199 to the 13-3-3/TAZpS89 complex; TCF199 is shown as orange surface. B-C) Detail of the two possible orientations of TCF199 within the binding site identified by MD simulations. 14-3-3 is shown as green cartoon, TAZpS89 is shown as magenta cartoon. In panels B-C, TCF199 is shown as cyan sticks, non-polar H atoms were omitted.

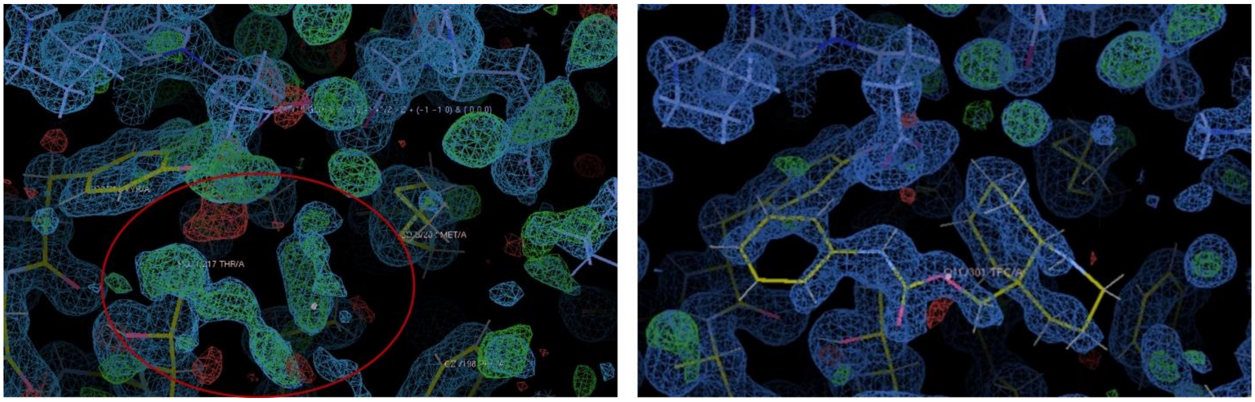

Figure S5: TCF199`s binding site analysis. Electron density before modeling (red circle; left-hand side) and after modeling (right-hand side).

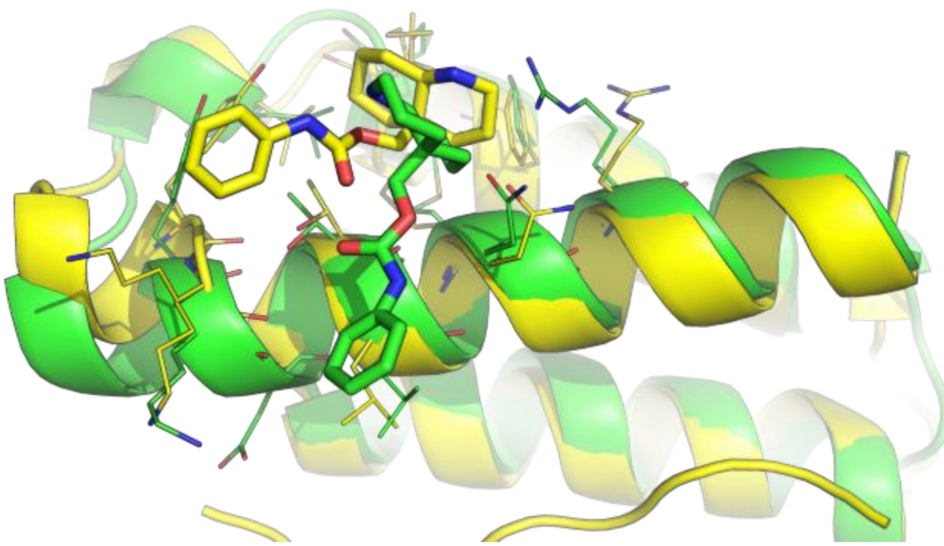

Figure S6: Comparison of MD vs X-ray structure. Structural superimposition between the most representative frame extracted from MD trajectories (in green) and the crystallographic structure (in yellow) of the 14-3-3/TAZpS89/TCF199 complex.

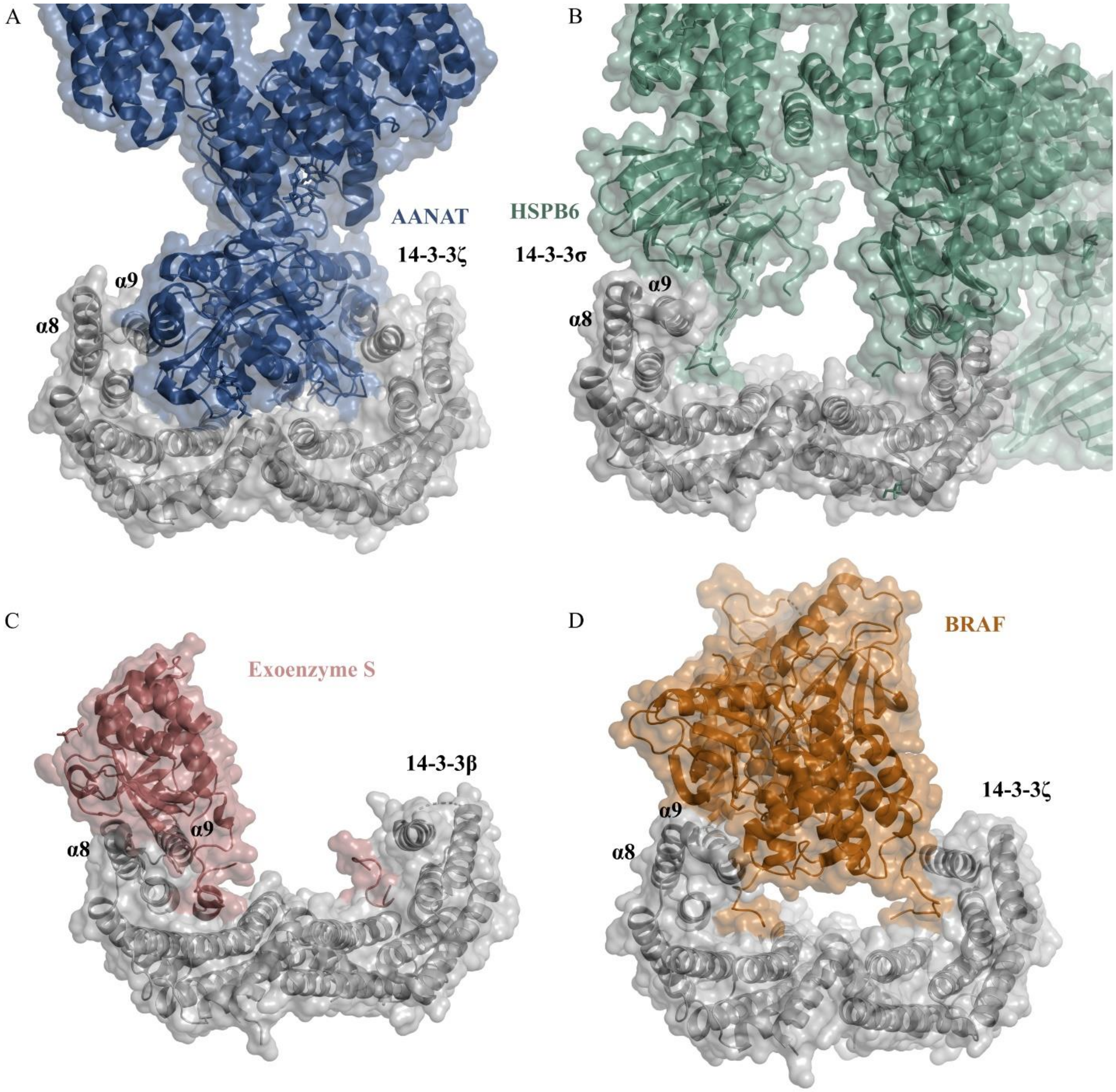

Figure S7: Representative structures of near full-length proteins in complex with 14-3-3. (A) Crystal structure of 14-3-3ζ in complex with AANAT (blue cartoon and surface) (PDB ID: 1IB1). (B) Crystal structure of 14-3-3σ in complex with HSPB6 (green cartoon and surface) (PDB ID: 5LTW). (C) Crystal structure of 14-3-3β in complex with Exoenzyme S (salmon cartoon and surface) (PDB ID: 6GN8). (D) Crystal structure of 14-3-3ζ in complex with BRAF (orange cartoon and surface) (PDB ID: 6U2H). 14-3-3 proteins are always depicted with white cartoons and surfaces.

Table S1-D: Data collection and refinement statistics.

	14-3-3σΔC/TAZ/TCF199
**PDB ID**	8R0Z
**Data collection**	
Wavelength (Å)	0.97626
Resolution (Å)	65.88 - 1.20
	(1.22 – 1.20)
Space group	C 2 2 21
Unit cell (Å)	81.48 111.98 62.75
Unique reflections	89660 (8853)
Multiplicity	12.8 (12.8)
Completeness (%)	100.0 (100.0)
Average I/σ	17.4 (4.5)
R-merge	0.075 (0.529)
R-meas	0.081 (0.572)
CC1/2	0.997 (0.940)
**Refinement**	
N° of protein/	1980/349/24
solvent/ligand atoms	
Rwork/Rfree (%)	16.95/17.82
RMSD Bond lengths (Å)	0.008
RMSD Bond angles (°)	1.20
Ramachandran favored (%)	98.30
Ramachandran allowed (%)	1.70
Ramachandran outliers (%)	0.00
Rotamer	0.47
outliers (%)	
Clashscore	2.51
Average Bfactor	15.87

Data collection statistics were calculated with Aimless and refinement statistics were extracted using the “table 1” tool of Phenix. Values in parenthesis correlate to high resolution shell.

Supplementary Methods

SD-Test. Denaturation test was performed for fragments TCF043 and TCF347. Three highest and three lowest fragment concentrations were selected and incubated with 14-3-3ζ/TAZpS89 for 1 hour in same buffer used for determining Kd values. Subsequently sodium dodecyl sulfate (SDS) and DTT were added to a final concentration of 2% and 20 mM and solution heated at 95 °C for 5 minutes while mixing. Afterwards samples were loaded into premium capillaries and placed into Monolith NT.115. Capillaries were scanned using RED detector.
